# Sebum and Hydration Levels in Specific Regions of Human Face Significantly Predict the Nature and Diversity of Facial Skin Microbiome

**DOI:** 10.1038/srep36062

**Published:** 2016-10-27

**Authors:** Souvik Mukherjee, Rupak Mitra, Arindam Maitra, Satyaranjan Gupta, Srikala Kumaran, Amit Chakrabortty, Partha P. Majumder

**Affiliations:** 1 BioMedical Genomics Centre, Kolkata, West Bengal, India; 2 Unilever R&D, Bangalore, Karnataka, India; 3 National Institute of Biomedical Genomics, Kalyani, West Bengal, India

## Abstract

The skin microbiome varies across individuals. The causes of these variations are inadequately understood. We tested the hypothesis that inter-individual variation in facial skin microbiome can be significantly explained by variation in sebum and hydration levels in specific facial regions of humans. We measured sebum and hydration from forehead and cheek regions of healthy female volunteers (n = 30). Metagenomic DNA from skin swabs were sequenced for V3-V5 regions of 16S rRNA gene. Altogether, 34 phyla were identified; predominantly Actinobacteria (66.3%), Firmicutes (17.7%), Proteobacteria (13.1%) and Bacteroidetes (1.4%). About 1000 genera were identified; predominantly *Propionibacterium* (58.6%), *Staphylococcus* (8.6%), *Streptococcus* (4.0%), *Corynebacterium* (3.6%) and *Paracoccus* (3.3%). A subset (n = 24) of individuals were sampled two months later. Stepwise multiple regression analysis showed that cheek sebum level was the most significant predictor of microbiome composition and diversity followed by forehead hydration level; forehead sebum and cheek hydration levels were not. With increase in cheek sebum, the prevalence of Actinobacteria (*p* = *0.001*)/*Propionibacterium (p* = *0.002*) increased, whereas microbiome diversity decreased (Shannon Index, *p* = 0.032); this was opposite for other phyla/genera. These trends were reversed for forehead hydration levels. Therefore, the nature and diversity of facial skin microbiome is jointly determined by site-specific lipid and water levels in the *stratum corneum*.

The resident microbiome is an essential component of the human skin with considerable inter-individual variations^1^,^2^,^3^. The microbiome composition varies by layer of the skin and changes with skin barrier disruption^4^,^5^. The skin microflora maintains a homoeostasis by cross-talk with the host innate immune system^6^,^7^,^8^. The skin also modulates microbial growth on its surface by the production of antimicrobial peptides (AMPs), thus protecting the host from infectious pathogens^9^. Dysbiosis in the skin microbiome often alters skin health and is linked to psoriasis, eczema, acne, atopic dermatitis and other similar disorders of the skin^10^,^11^,^12^,^13^,^14^. Past studies have provided evidence of some intrinsic factors in the human that modulate microbial density and types of microorganisms found in specific sites of the human body^15^,^16^. However, these factors and interactions are incompletely understood. Some characteristic features of the facial skin, such as concentration of sebum and level of hydration, are known to vary across individuals and across different areas of the face even within an individual. Here, we sought to determine whether observed differences in the nature and diversity of the facial skin microbiome associates with overall differences in characteristic features of the facial skin or with skin characteristics of specific areas of the face. Further, we have sought to understand the temporal stability of the nature and diversity of the facial skin microbiome.

## Results

### Sebum and Hydration Levels from Forehead and Cheek Regions

The sebum concentration of forehead (mean ± s.e.m. = 96.17 ± 12.29 μg/cm^2^) was significantly higher (paired T-test *p*-value = 4.0 × 10^−6^) than that of cheek (mean ± s.e.m. = 38.12 ± 4.07μg/cm^2^) (Table 1). For hydration level, such difference between forehead and cheek was not observed (*p* = 0.302) (Table 1). Levels of sebum and hydration were significantly and positively correlated both on the forehead (r = 0.47, *p*-value = 0.011) (Supplementary Fig. 1a) and on the cheek (r = 0.51, *p*-value = 0.004) (Supplementary Fig. 1b). Hydration levels, but not sebum levels, on both forehead (*p* = 0.023) and cheek (*p* = 0.042) varied significantly with age.

### Sequence Reads: Data Quality and Processing Results

A total of 1,700,608 sequence reads spanning the V3-V5 region of the heterogeneous mixture of 16S rRNA gene were generated for 29 out of the 30 samples (See Materials and Methods for details). These were subjected to initial quality control filters for minimum read length, number of ambiguous bases, quality value per base and homopolymers. On an average, (95.7 ± 0.97)% of reads were retained per sample after filtering (Supplementary Table 1). The average length of amplicon varied between 511–530 bases; (mean ± s.d. = 523.52 ± 5.09). The total number of reads after initial quality filtering, homopolymer and chimera removal was 1,531,090; the number of sequence reads for most samples exceeded 30,000 (Supplementary Table 1). The percentage of chimeric sequences among individuals varied between ~1% to ~10% (mean ± s.d. = 4.88 ± 3.77).

### Abundance and Diversity of the Skin Microbiome: Inter-Individual Variation

**Figure 1:**
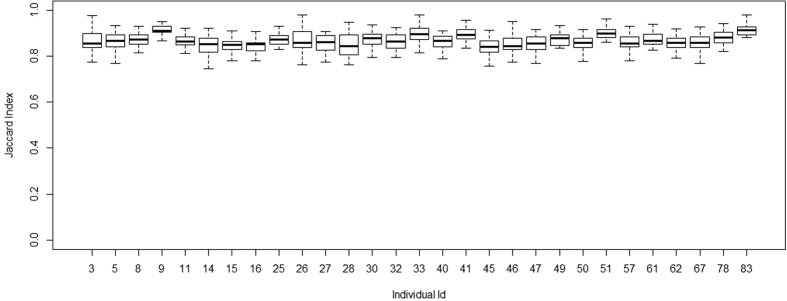
Estimation of β-Diversity: Box-Plots of Jaccard Index for all individuals.

**Figure 2:**
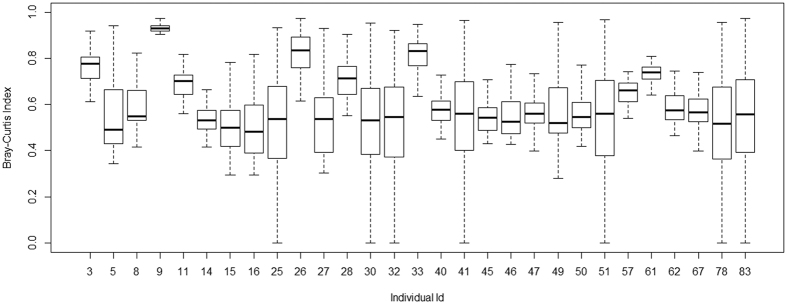
Estimation of β-Diversity: Box- Plots of Bray-Curtis Dissimilairty Index for all individuals.

The quality filtered and non-chimeric sequences for all the samples were clustered into bins with pairwise sequence similarity of ≥97% between any two randomly chosen reads. The total number of such clusters – Operational Taxonomic Units (OTUs) – was found to be 34,567 upon removal of singletons (OTUs with only one sequence read in one individual and absent in all the other individuals). The sequences were rarified by subsampling with minimum number of reads (~20000 reads) among all the 29 samples and plateaued rarefaction curves were observed (Supplementary Fig. 2). Diversity indices (Shannon and Chao indices) also varied substantially among the study participants (Table 1) and were significantly correlated (r = 0.91, *p* < 0.01) (Supplementary Fig. 3). After subsampling, the total number of OTUs among all samples was 28076. We note that even though the number of OTUs per sample, after subsampling, varied from 1202 to 5697 (Supplementary Fig. 4), this variation was not a reflection of the nature of the data generated among the samples. The correlation between the number of OTUs and the total number of non-chimeric sequence reads per sample (Supplementary Fig. 5) was r = 0.28 (p > 0.05). The inter-individual variability was estimated as a function of both community membership (presence/absence of shared OTUs) and community structure (presence/absence of shared OTUs coupled with difference in relative abundances) by Jaccard and Bray-Curtis indices, respectively. The community membership, as average estimates of Jaccard index, ranged from 0.84 to 0.92 across individuals (Fig. 1), while the Bray-Curtis dissimilarity index estimates showed wider inter-individual variation ranging from 0.52 to 0.93 (Fig. 2), except for sample number 9 which was highly dissimilar to all the other samples (details about this sample are described in the next section). Hence, the community membership, but not the community structure, of the facial skin microbiome was generally conserved.

### Taxonomic Classification

The skin microbiome data were classified into bacterial taxa, from phyla to genera, with high bootstrap confidence (~80%). This was done by aligning the total number of reads (after removal of chimeric sequences) to the bacterial databases, Greengenes and Silva Gold, using QIIME (v.1.8.0) and Mothur (v.1.33.3) with and without subsampling, respectively (details provided in Materials and Methods); the results obtained by both methods correlated significantly well both at the levels of phylum (p < 0.05) (Supplementary Fig. 6a,b) and genus (p < 0.001) (Supplementary Fig. 7a,b).The number of reads assigned to each taxon was normalized to percentage proportions, based on the total number of reads for a particular sample. A total of 26 phyla (accounting for an average of ~99% of the total sequence reads) and over 450 genera (accounting for an average of ~89% of the total sequence reads) were commonly identified by Mothur and QIIME. The difference in average relative abundances between the phyla and genera levels can be attributed to the large number of taxa that were unclassified till the genus level in both Mothur and QIIME. However, except for Streptophyta (~2.5%) (phylum: Cyanobacteria) that was classified till the genus level by Mothur and till the order level by QIIME, each of the uniquely identified (Total Relative Abundance: Mothur = 1.5%, QIIME = 0.7%) and unclassified (Total Relative Abundance: Mothur = 7.3%; QIIME = 8.0%) genera had an average relative abundance of <0.1% by both the classifiers (Supplementary Table 2). For further downstream analyses, we selected the QIIME pipeline. After removing singletons, 34 phyla were identified; predominantly Actinobacteria (64.6%), Firmicutes (17.3%), Proteobacteria (12.7%) and Bacteroidetes (1.4%). About 1000 genera were identified; predominantly *Propionibacterium* (57.1%), *Staphylococcus* (8.5%), *Streptococcus* (3.9%), *Corynebacterium* (3.5%) and *Paracoccus* (3.2%). In the previous section, the average Bray-Curtis index plot showed that sample number 9 was dissimilar to all the other 28 samples. For that analysis, all the OTU clusters are considered to be equally different. However, in the bacterial communities, there is phylogenetic relatedness between some community members compared to others. To incorporate this community relatedness in assessing inter-individual variability, we next performed Weighted UniFrac analyses by incorporating phylogenetic informations from the taxonomic classifications data. The results of the Weighted UniFrac analysis also showed that sample number 9 was an outlier (Supplementary Fig. 8)^17^. On close scrutiny of the taxonomic data, this individual was found to harbor an unusually high frequency of Cyanobacteria (~50%) compared to the average frequency of 2.5% in the remaining samples. Cyanobacteria on healthy skin is usually considered to be a contaminant, because of its sequence similarity to chloroplast DNA sequence, sometimes caused by use of herbal skin care products^18^. This volunteer, however, did not report use of any such skin care product. For further analysis, we removed cyanobacteria from our data and normalized the data by the relative abundance of cyanobacteria for all the samples. The relative abundances of the major phyla after normalization were: Actinobacteria (66.3%), Firmicutes (17.7%), Proteobacteria (13.1%) and Bacteroidetes (1.4%); not significantly different from the abundances noted before normalization. At the genus level, seven genera accounted for more than 80% of the sequence reads: *Propionibacterium* (58.6%), *Staphylococcus* (8.6%), *Streptococcus* (4.0%), *Corynebacterium* (3.6%), *Paracoccus* (3.3%), *Neisseria* (1.5%) and *Acinetobacter* (1.3%).

### Sebum and Hydration Levels Significantly Predict Skin Microbiome Patterns

**Figure 3:**
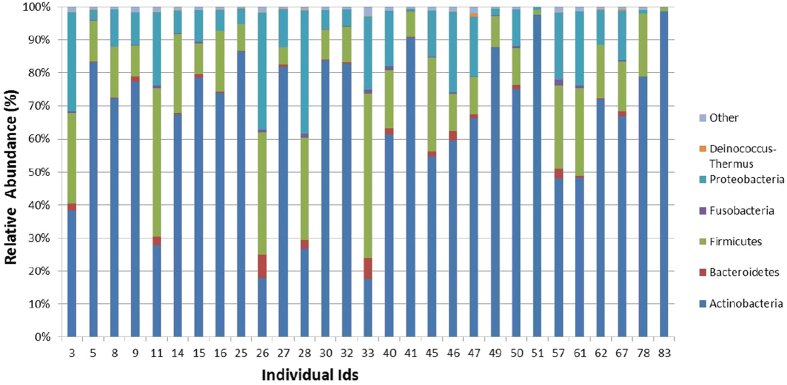
Distribution of Phyla with frequency ≥1% of reads in at least one among the 29 female volunteers recruited in the study.

**Figure 4:**
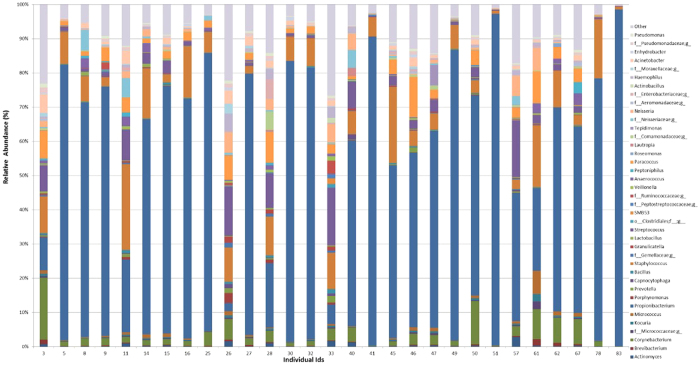
Distribution of Genera with frequency ≥1% of reads in at least one among the 29 female volunteers recruited in the study.

We sought to test whether sebum and hydration levels from different facial regions significantly predict the nature and diversity of facial skin microbiome. For this, we included only those taxa for further analysis that had a relative abundance ≥1% in at least one among the 29 individuals. Six phyla (Fig. 3) (Actinobacteria, Firmicutes, Proteobacteria, Bacteroidetes, Fusobacteria and Thermi or Deinococcus-Thermus) and 39 genera (Fig. 4) satisfied this criterion; all other taxa were grouped as “Other”.

Stepwise multiple regression analysis showed that the levels of sebum on cheek and hydration on forehead were significant predictors of the relative abundance of specific phyla – Actinobacteria (*p* = 0.001), Proteobacteria (*p* = 0.028), Firmicutes (*p* = 0.001) and Bacteroidetes (*p* = 0.003); and genera – *Propionibacterium (p* = 0.002), *Haemophilus (p* = 0.004), *Enhydrobacter (p* = 0.023), *Granulicatella (p* = 0.002), *Streptococcus (p* = 0.003), *Veillonella (p* = *0.027*), *Bacillus (p* = *0.012*) and *Prevotella (p* = *0.017*) (Table 2). The most significant observation is the differential predictive potential of sebum and hydration levels from specific facial regions. Sebum level on cheek was a significantly stronger predictor of skin microbiome composition and diversity whereas forehead sebum level was not, but in respect of hydration, the level on the forehead, but not on the cheek, was the significant predictor.

The proportion of the phylum Actinobacteria (*p* = 0.001) and the genus *Propionibacterium (p* = 0.002) significantly increased with increasing concentration of sebum on the cheek, while the proportions of the other phyla/genera decreased. The diversity and richness of the skin microbiome decreased with increase in oilyness of the cheek (Shannon Index, *p* = *0.032*), (Chao Index, *p* = *0.001*) (Table 2). Exactly the opposite trend was observed for the forehead hydration level. The proportion of variance explained by Cheek Sebum ranged from 24.12% for Actinobacteria to 16.71% for Proteobacteria at the phylum level and 21% for *Bacillus*, 20.35% for *Propionibacterium* to 13.97% for *Haemophilus* at the genus level (Table 2). The proportion of variance explained by Forehead Hydration levels were 15.09% for Actinobacteria and 12.27% for Bacteroidetes at the phylum level and 20.72% for *Haemophilus* to 13.62% for *Propionibacterium* at the genus level (Table 2).

### Temporal Stability of the Skin Microbiome

We also assessed the temporal stability of the skin microbiome profiles in order to understand the differences in distribution patterns with change in environmental conditions over time^1^,^3^. For this, we compared the microbiome profiles in a subset of the healthy female volunteers (n = 24) after a follow-up period of two months (Table 3). A phylum or a genus was included for analyses only if there was at least 1% reads corresponding to this phylum/genus in at least one among the 29 individuals. Except *Paracoccus* *(p* < *0.001)*, all the phyla and genera (Table 3) showed temporal stability, at least over the period of two months, indicating that these taxa may constitute the core facial microbiome.

### Comparison with Skin Microbiome Profiles in Other Populations

To understand whether the healthy skin microbiome harbors any population specific patterns, we compared the relative abundance of skin microbiome at both the levels of phylum and genus observed in our study with studies from The Netherlands (n = 5)^5^ and China (n = 40)^19^. In our study, the number of unique OTUs after singleton removal was found to be 7352 (21.3% of total). (Unique OTUs are those that are found in only one individual whereas singleton OTUs are those that contain only one read in only one individual.) This is similar to that found in the study performed by Leung *et al.*^19^ in Chinese populations, where the number of unique OTUs, after singleton removal, from forehead was 6435 (22.6% of total). The four most abundant phyla found in our study – Actinobacteria, Proteobacteria, Firmicutes and Bacteroidetes – were the same as those found in the Chinese study. The major genera identified in the Chinese study were: *Propionibacterium*, *Staphylococcus*, *Acinetobacter*, *Streptococcus*, *Enhydrobacter* and *Corynebacterium*; these were also major genera in our study, except for *Enhydrobacter* for which relative abundance was <1%. When the relative abundances of major phyla and genera identified in our study were compared with the study performed by Zeeuwen *et al.*^5^ involving healthy individuals from The Netherlands, significant differences were observed (Supplementary Table 3) in the proportions of (a) phylum Proteobacteria (India: 13.1%; The Netherlands: 0.4%), and (b) genera *Corynebacterium* (India: 3.6%; The Netherlands: 7.7%) and *Streptococcus* (India: 4.0%; The Netherlands: 0.8%).

The results of our study and the Chinese study (Leung *et al.*^19^) showed remarkable similarities although different variable regions were sequenced. We have sequenced the V3-V5 region using the GS-FLX platform, while Leung *et al.*^19^ have sequenced the V4 region using the MiSeq platform. DNA isolation and sample preparation kits used in the two studies were also different. However, the results of our study were considerably different from those obtained from The Netherlands (Zeeuwen *et al.*^5^), although similar regions were sequenced using identical sequencing platforms and similar methodologies were adopted for DNA isolation and sample preparation. Therefore, concordance or discordance of results is not due to similarities or differences in methodologies and platforms.

The population specific patterns identified in studies performed in India (our study), The Netherlands and China provide evidence of reference skin microbiome being different across populations. This reinstates the need for undertaking more such studies, especially in non-western populations with diverse population histories.

## Discussion

Data of studies^1^,^15^,^16^, conducted primarily among Caucasians, have characterized microbiome signatures from different skin sites and have documented considerable topographical and temporal variance across dry, moist and sebaceous skin sites. To the best of our knowledge, such data are, however, lacking from the ethnically distinct Asians, except for a study conducted among Chinese individuals^19^. The present study fills this gap. Here, we have characterized the nature and diversity of the facial skin microbiome of healthy volunteers of Dravidian descent residing in Bangalore, India. We have also correlated inter-individual differences in microbiome profiles with differences in their sebum and hydration levels on forehead and cheek regions. Consistent with results of some previous studies^20^,^21^,^22^,^23^, skin hydration level varied significantly with age; no significant variation with age was found for sebum concentration on either forehead or cheek^22^,^23^. These findings appear to be consistent across ethnicities^21^,^22^,^23^,^24^,^25^,^26^. We note that some previous studies have reported lack of significant relationship of hydration with age^27^ and significant relationship of sebum with age^21^,^27^. One reason for lack of uniformity in results is difference in ages of individuals included in these studies; sebum concentration is known to be dependent on hormonal levels which vary by age group^24^,^25^.

The proportions of the most abundant phyla estimated from the present data were similar to those found in the Western populations^1^: Actinobacteria (India: 66.3%; USA: 51.8%), Firmicutes (India: 17.7%; USA: 24.4%), Proteobacteria (India: 13.1%; USA: 16.5%) and Bacteroidetes (India: 1.4%; USA: 6.3%). However, when data on only facial skin microbiome were compared, significant differences in the proportions of the phylum Proteobacteria (India: 13.1%; The Netherlands: 0.4%), and genera *Corynebacterium* (India: 3.6%; The Netherlands: 7.7%) and *Streptococcus* (India: 4.0%; The Netherlands: 0.8%) were observed^5^. The majority of skin microbes populate the *stratum corneum*, the outermost layer of the skin. *Stratum corneum* has a thin texture in the facial region with some unique functional characteristics to provide hydrated skin surface with relatively poor barrier function. However, there are differences in properties of the *stratum corneum* and also of biophysical properties on the forehead, cheek, nose and perioral regions^26^,^28^. The facial regions are more sebaceous and moist than the trunks and limbs^28^,^29^. The forehead is more sebaceous than the cheek^26^. The T-zone of the face, comprising the forehead and nose, has previously been reported to have higher levels of sebum than other facial regions^28^,^30^.

In past studies, *Propionibacterium* was reported to be the most abundant bacterium in the sebaceous sites and *Corynebacterium* was the most abundant in the moist sites, both followed by *Staphylococcus*. The dry sites were reported to have a mixed population^1^,^15^,^16^. We have found that the sebum concentration of the cheek, not the forehead, is significantly correlated with microbial composition and diversity. This can be explained by the fact that generally forehead sebum levels are always much higher than the cheek sebum levels, hence changes in forehead sebum levels probably do not alter the sebaceous nature of the site to significantly impact on bacterial abundances, which is more pronounced in the cheek region. *Propionibacterium* was the most abundant genus whose frequency increased with increase in cheek sebum levels. OTU heatmap (Supplementary Fig. 9) generated with representative sequences from ~34,000 OTUs identified *Propioinibacterium acnes* to be the most abundant species. *P. acnes* is a commensal bacterium that binds to oleic acid of the sebum, which in turn facilitates their co-aggregation in sebaceous sites and metabolizes fatty acids and other sebaceous fluids to propionic and acetic acid that help in eradication of harmful microbes^31^. Some strains of *P. acnes* are, however, associated with *acne vulgaris* which is a common inflammation of the skin. *Propionibacterium* also synthesizes proteases which have detrimental effect on other commensal and/or pathogenic strains in the skin of that individual. This is consistent with negative correlation of microbial diversity and richness indices (Shannon Index and Chao Index) with increase in cheek sebum level. The opposite scenario is favoured when the skin hydration increases and the skin becomes moist helping the other kinds of skin commensals to grow. Our study has shown that this correlation is also associated with changes in abundances of specific bacterial taxa. The hydration level on the forehead, rather than on the cheek, was found to be the next best predictor of nature and diversity of facial skin microbiome. The cheek is generally considered to be more hydrated in young females^32^. Therefore, change in cheek hydration might not alter the moisture content significantly enough to impact on the change in bacterial abundances, which might be more pronounced for changes in forehead hydration levels.

We did not find any association of *Corynebacterium* or *Staphylococcus* with either sebum or hydration levels of the facial skin; instead we found *Streptococcus* and *Haemophilus* to be significantly associated with both sebum and hydration levels on cheek and forehead, respectively. *Corynebacterium* is a nitrogen fixing coryneform that hydrolyzes urea from sweat to ammonia which serves as a nitrogen source for other cutaneous microbes and helps in maintenance of skin health^33^. The significant association of *Streptococcus* and *Haemophilus*, rather than *Corynebacterium* with change in sebum and hydration levels in the healthy skin typical to our study population might have an impact on the overall skin health since *Corynebacterium* is a nitrogen fixing commensal and *Streptococcus* and *Haemophilus* are often linked to skin infections^34^.

Considerable temporal variability has been observed by Flores *et al.*^35^ in the microbiome community membership and structure, prompting the proposal of a personalized pattern of such temporal variations. On the contrary, we have found that the microbiome patterns showed considerable temporal stability at both the phylum and genus level, except for the genus *Paracoccus* *(p* < *0.001)*. None of the other phyla or genera tested showed significant temporal variations at least for a period of two months.

Except for *Propionibacterium* and *Streptococcus*, all the other genera that were significantly associated with variations in sebum and hydration levels and yet maintained temporal stability over time, belong to rare taxa. It has been recently argued that rare taxa help in maintaining an overall microbial community structure^36^. Here, we have also shown that the abundances of these rare taxa are influenced by host factors, indicative of functional importance of these taxa.

Thus, the differences in composition, diversity and temporal variability of the skin microbiota between Asian (this study) and Western populations are considerable; similar to the findings of the gut microbiota for Africans^37^. This may have implications in understanding differences in prevalence of various skin conditions, such as acne, psoriasis, dermatitis etc. Our findings provide the first direct evidence that quantitative levels of sebum and hydration in specific regions of facial skin are significantly associated with the distribution of specific bacterial taxa inhabiting the *stratum corneum*.

## Methods

### Study Design and Sample Collection

This study was approved by the Institutional Ethics Committees of the National Institute of Biomedical Genomics, West Bengal, and Unilever Research and Development Center, Bangalore, India. The study was performed in accordance with and following the Declaration of Helsinki Principles. Sample collection and all subsequent experimental procedures described in the Materials and Methods section were conducted in accordance with relevant guidelines and regulations.

A cohort of 30 healthy female individuals was recruited (mean age: 29.5 ± 5.48 years) with written, informed consent for participation in the study from in and around Whitefield area of Bangalore city, India. No study participant had taken any antibiotic for the past 6 months. Each study participant was advised not to wash her face with soap or take a bath at least 12 hours before arrival in the sample collection site. On arrival, their faces were washed with sterile water (MilliQ) and they were put in a controlled environment at 24 °C and relative humidity of 45% for a minimum period of 4 hours before sample collection. This was done to provide sufficient time for the resident skin microflora and the levels of skin health parameters like sebum and hydration, to regain their individuality. Using Sebumeter (measures sebum content, Courage + Khazaka electronic GmbH) and Corneometer (measures hydration, Courage + Khazaka electronic GmbH), two readings on levels of sebum and hydration from forehead and two readings from the cheek regions were taken on each study participant for measuring sebum and hydration levels. One individual showed extremely low values for both forehead and cheek sebum even on repeated measurements; hence we excluded her data from further analysis. After a period of 14 days, the study participants were again recalled and the same protocol was implemented on each. During the 14-day period between the two phases of sample collection, each study participant was advised to use a specific marketed soap which does not have any antibacterial active in it. They were also advised not to use any skin care product on the facial regions for the 14-day period of time. Skin swabs were collected from facial regions, excluding lips and nose, using moistened sterile pads (2 × 2 cm) for 10 seconds each. Buffer used for sample collection was 1X Phosphate Buffer Saline (PBS) pH 7.0 + Tween 80 (0.5%). We ensured that the volunteers covered their entire face (except for the region around mouth and nose) with moist sterile pads. The sterile pads were then put in sterile tubes containing 10ml of 1X PBS buffer. The tube was vortexed for 5 minutes to ensure that all microbes get resuspended into the buffer. The supernatant was then centrifuged for 15 minutes at 15000g and the microbial pellet was separated. Finally, genomic DNA was isolated from the microbial pellet following manufacturer’s protocol (BiOstic Bacteremia DNA Isolation Kit, MO BIO Laboratories Inc). As a negative control, we also included a swab pad exposed to the sample collection room for 10 minutes following which we put it in PBS buffer and processed for microbial DNA isolation using exactly the same protocol as is performed for skin swab samples. Using V3 primer sequences we amplified four skin swab DNA samples, isolated following the same protocol as listed above, along with the elution product obtained from the similarly processed negative control cotton swab. However, on gel electrophoresis, we did not find any detectable band for the negative control although we obtained bands for all the other four skin swab samples with desired fragment sizes (Supplementary Fig. 10). As a standard, we have also included a PCR mix negative control which also did not show any band after gel electrophoresis. Hence, we did not proceed to sequence the cotton swab negative control any further. This approach has been used previously (Zeeuwen *et al.*)^5^. Efforts were made to collect skin swabs from the same set of individuals after a period of 60 days; this was possible for 24 of the 30 individuals.

We would like to point out that we actually tried to collect skin swabs from specific areas of the face, just as we collected sebum and hydration levels from specific areas of the face, and carry out deep-sequencing of 16S rRNA genes from these skin swabs. However, this was not possible, since the amount of microbes collected in a single skin swab was too low for DNA extraction for deep-sequencing. We, therefore, created a pool of microbes from the skin swabs collected from different regions of the face for deep-sequencing. We also note that the forehead has very high sebum level per unit area compared to the cheek in an individual. Therefore, small perturbations in sebum level in the forehead are unlikely to impact on the nature and abundance of microbes, while such perturbations in the cheek are more likely to be impactful.

### Massively Parallel Multi-tagged Pyrosequencing

The microbial DNA isolated from skin swabs of healthy volunteers were sequenced for the variable region V3-V5 of the 16S rRNA gene for bacterial classification. Multiplexed massively parallel sequencing was performed in GS-FLX (Roche) with amplicons generated by PCR with universal primers 357F (5′-CCTACGGGAGGCAGCAG-3′) and 926R (5′- CCGTCAATTCMTTTRAGT-3′) specific for the V3-V5 region tagged with individual specific multiplex ids (MIDs). Prior to sequencing, purified genomic DNA isolated from skin swabs were quantitated in NanoDrop Spectrophotometer (Thermo Scientific) and Qubit fluorometer (Invitrogen). A 570 bp amplicon, spanning V3-V5 region of 16S rRNA, was PCR amplified from 2μl of genomic DNA per sample using fusion primers 357F (5′-CCTACGGGAGGCAGCAG-3′) and 926R (5′- CCGTCAATTCMTTTRAGT-3′) with MID sequences^37^, Fast Start Hifidelity PCR System (Roche) and PCR cycle and other conditions of 94 °C for 3 minutes, 35 cycles of 94 °C for 15 seconds, 55 °C for 45 seconds and 72 °C for 1 minute, followed by hold at 72 °C for 8 minutes and 10 °C until stop, were used. Amplicons were viewed by 2% agarose gel electrophoresis and SYBR Gold (Invitrogen) staining. Both positive and negative controls were included in the 16S rRNA PCR amplification process along with the samples followed by gel electrophoresis where no visible bands were observed for the negative control as opposed to the positive control and the samples where clear bands were observed at desired amplicon length (~600 bp). Amplicons were purified from gel slices using MinElute Gel Extraction kit (Qiagen), quantitated in Qubit fluorometer (Invitrogen) and viewed in high sensitivity DNA chips in 2100 Bioanalyzer (Agilent). Amplicons from 14 samples were pooled per sequencing run (7 samples in each region of a two region Pico-titre plate). Sequencing was performed using GS FLX platform (Roche) by Titanium chemistry with primer 926R as the sequencing primer following HMP Skin microbiome study protocol^16^.

### Metagenomic Data Analysis

For each of the sequencing runs, the sequence flowgram format files (SFF) were converted to FASTQ files and FASTQC reports were generated (using the online tool available at: http://www.bioinformatics.babraham.ac.uk/projects/fastqc). The initial quality control was performed based on the FASTQC reports for each data file. The files were then demultiplexed for the MID sequences and trimmed using the following filter criteria: Number of Ambiguous bases = 0–2, QV per base = 20–25, Minimum sequence length = 150–200, Maximum Sequence Length = 650–700 and Number of Homopolymers = 4–6 based on the nature of the data. The metagenomic data analysis was carried out using RDP (Release 11) (for initial QA/QC), Mothur (v.1.33.3) and QIIME (v.1.8.0) for cross-validation (results were mostly congruent)^39^,^40^,^41^. In QIIME, default criteria were used for analysis and subsampling was done for the minimum number of reads among all the 29 samples (~20000 reads). The chimeric sequences were removed using UCHIME and the alignments were done with Greengenes and SILVA GOLD databases for QIIME and Mothur, respectively. The sequence reads were clustered into distinct Operational Taxonomic Units (OTUs) based on pairwise sequence similarity among all the sequences with a threshold of 97% for equivalence to genus level clustering^8^,^16^,^42^. The numbers of OTU counts for each of the samples were estimated and rarefaction plots were constructed to examine whether the microbiome diversity estimated from the sequence data of each individual reached a plateau. The Shannon and Chao Indices and Phylogenetic Diversity were estimated and rarefaction plots were generated. Shannon Index is an estimator of microbial diversity whereas Chao index is an estimator of richness of taxa per individual. The Jaccard and Bray-Curtis Dissimilarity indices were estimated for all the individuals studied. The actual number of sequence reads aligned with each taxon per individual was normalised by the total number of reads of that individual and percentage proportion was calculated for each hierarchical level of taxonomic classifications from Phylum to Genus.

### Statistical Analysis

We have included data on only those taxa (phyla and genera) that were represented by a frequency of ≥1% of reads in at least one individual. All the other taxa were pooled into a separate group called “Other”. Statistical analysis was performed using SPSS, R and several in-house programs developed by us. Sebum and hydration levels from forehead and cheek were estimated for each individual and correlated with i) bacterial abundance and ii) alpha diversity indicators (Shannon Index, Chao Index, Number of OTUs and Number of Genera per sample). We reasoned that it may be possible for the abundance and diversity indicators to be influenced by sebum and/or hydration levels of only a subset of the sites from where skin swabs were taken. The stepwise regression model thus can provide additional information on which of the four variables provided statistically significant prediction when correlations among the predictor variables were taken into account. Therefore, a forward stepwise multiple regression analysis was performed to identify the significant predictors of facial microbiome characteristics from among sebum and hydration levels on cheek and forehead. Of course, the full model with all independent variables was found to be significant if one or more independent variables were identified to be significant in the stepwise analysis. If no significant predictor was identified in the stepwise analysis, then the full model was statistically non-significant. In the stepwise regression analysis, abundance or diversity indicator was used as the dependent variable and sebum and hydration levels of forehead and cheek (four variables) were used as independent variables. A constant was included in the regression equation. The probability of the F-ratio was used for entry/removal of a dependent variable; the probability values of F used for entry and removal were 0.05 and 0.10, respectively, which are the default values in SPSS. The proportion of total variance explained by a forward stepwise regression model comprising one or more predictors was estimated as the ratio of the Model Sum of Squares (SSM)/Total Sum of Squares (SST); when more than one predictor was involved, estimates for individual predictors were obtained by subtraction using the same order as the stepwise entry of predictors into the regression model. For examining the temporal differences in facial microbiome characteristics, paired t-tests (cross-validated by the non-parametric Wilcoxon Signed Rank Test, data not shown) were done for each phylum and genus with read frequency ≥1%.

## Additional Information

**Publisher's note**: Springer Nature remains neutral with regard to jurisdictional claims in published maps and institutional affiliations.

**How to cite this article**: Mukherjee, S. *et al.* Sebum and Hydration Levels in Specific Regions of Human Face Significantly Predict the Nature and Diversity of Facial Skin Microbiome. *Sci. Rep.* **6**, 36062; doi: 10.1038/srep36062 (2016).

## Supplementary Material

Supplementary Information

## Figures and Tables

**Table 1:** Sebum-Hydration levels on forehead and cheek along with the alpha diversity estimates of facial skin microbiome.

Sample ID	Age	Sebumeter Readings (Measures Sebum)		Corneometer Readings (Measures Hydration)		OTU Count	No. of Genera	Shannon Index	Chao Index
		Forehead Sebum^1^ (μg/cm^2^)	Cheek Sebum^1^ (μg/cm^2^)	Forehead Hydration^2^ (a.u.)	Cheek Hydration^2^ (a.u.)				
3	29	54.00	12.00	43.50	42.00	4818.00	538.00	9.70	9356.50
5	28	127.50	53.50	61.50	64.00	2572.00	255.00	5.62	5679.00
8	35	242.00	56.50	58.50	60.50	2978.00	287.00	6.84	6264.60
9	26	71.50	55.50	49.00	56.50	2352.00	282.00	6.75	4835.52
11	34	200.50	38.00	60.50	67.50	3695.00	477.00	7.79	8273.61
14	28	168.50	49.00	63.50	56.00	3579.00	316.00	7.53	7158.28
15	26	79.00	54.00	53.00	66.50	3504.00	354.00	6.40	7472.62
16	25	30.50	1.50	43.50	43.50	2972.00	381.00	5.88	6318.96
25	31	29.00	19.50	43.00	52.00	2048.00	227.00	4.30	4447.22
26	31	60.00	19.00	65.50	59.00	5086.00	490.00	9.97	9642.07
27	28	101.00	43.50	65.50	52.00	2930.00	384.00	5.65	6449.29
28	30	65.50	21.50	55.00	58.50	4328.00	426.00	9.14	8369.33
30	30	30.00	38.00	45.00	46.50	2063.00	233.00	4.00	4766.82
32	30	31.50	8.00	41.00	36.00	2422.00	411.00	4.26	5765.17
33	25	52.00	3.50	47.00	40.00	5697.00	694.00	9.98	12035.56
40	32	63.50	23.00	57.50	66.50	3908.00	276.00	8.07	7599.19
41	32	31.00	25.00	46.50	45.00	1320.00	194.00	2.94	2955.63
45	30	67.00	14.00	48.50	34.50	3831.00	435.00	7.08	8009.39
46	26	64.00	15.00	42.50	27.50	3893.00	549.00	7.15	8784.03
47	27	136.50	60.50	58.50	63.00	4584.00	367.00	8.17	9229.35
49	31	269.00	56.50	63.00	52.00	2318.00	201.00	5.75	5132.09
50	30	64.50	63.00	53.00	60.50	4065.00	397.00	7.79	8566.01
51	30	185.00	78.00	48.50	59.50	1512.00	138.00	4.59	3032.90
57	23	46.50	29.50	49.00	35.00	4733.00	497.00	8.78	9706.82
61	34	126.00	55.50	60.50	51.50	4504.00	375.00	9.42	8514.84
62	35	60.00	24.00	63.00	53.00	4115.00	355.00	8.04	8477.21
67	31	50.50	60.50	56.50	55.00	4327.00	372.00	8.18	8393.79
78	32	114.50	58.50	61.00	54.00	1693.00	194.00	4.12	3618.66
83	27	168.50	69.50	41.50	40.00	1202.00	68.00	4.29	2029.98
**Mean**	**29.52**	**96.17**	**38.12**	**53.26**	**51.64**	**3346.52**	**350.79**	**6.83**	**6927.05**
**SEM**	**0.58**	**12.29**	**4.07**	**1.51**	**2.00**	**226.56**	**25.34**	**0.37**	**443.48**

^1^Comparison between Forehead and Cheek Sebum is significant (paired T-test *p*-value = 4.0 × 10^−6^).

^2^Comparison between Forehead and Cheek Hydration is not significant (*p* = 0.302).

**Table 2:** Results of stepwise multiple regression analysis: Skin health parameters that significantly predict proportions of various bacterial communities and microbial diversity indicators.

Phylum/Diversity Indicator	*Genus*	Cheek Sebum (μg/cm^2^)			Forehead Hydration [Age adjusted] (a.u.)		
		Regression Coefficient (% of Total Variance explained)	Std.Error	p-value	Regression Coefficient (% of Total Variance explained)	Std.Error	p-value
Actinobacteria	***All genera within phylum***	0.657 (24.12)	0.17	0.001	−1.307 (15.09)	0.503	0.015
	Propionibacterium	0.735 (20.35)	0.213	0.002	−1.459 (13.62)	0.63	0.029
Proteobacteria	***All genera within phylum***	−0.193 (16.71)	0.083	0.028	*		
	Haemophilus	−0.029 (13.97)	0.009	0.004	0.077 (20.72)	0.027	0.008
	Enhydrobacter	−0.005 (17.79)	0.002	0.023	*		
Firmicutes	***All genera within phylum***	−0.34 (22.61)	0.091	0.001	0.665 (14.6)	0.271	0.021
	Granulicatella^1^	−0.012 (15.24)	0.004	0.002	*		
	Streptococcus	−0.127 (18.30)	0.039	0.003	0.265 (13.93)	0.115	0.029
	Veillonella	−0.006 (16.83)	0.003	0.027	*		
	Bacillus	−0.007 (21.00)	0.002	0.012	*		
Bacteroidetes	***All genera within phylum***	−0.047 (19.79)	0.014	0.003	0.09 (12.27)	0.042	0.04
	Prevotella	−0.01 (19.44)	0.004	0.017	*		
DIVERSITY INDICATORS^2^	**Shannon Index (6.83 ± 0.37)**	−0.037 (14.12)	0.016	0.032	0.139 (14.14)	0.048	0.007
	**Chao Index (6927.05 ± 443.48)**	−63.407 (16.86)	17.778	0.001	161.413 (22.09)	52.621	0.005
	**Total No. of Genera (350.79 ± 25.34)**	−4.493 (37.03)	0.95	0.000069	6.075 (9.58)	2.812	0.04

*Variable not significant.

^1^Cheek Hydration is a significant (p = 0.014) predictor with regression coefficient 0.021 ± 0.008 (17.85).

^2^Figures in parentheses are values of mean ± s.e.

**Table 3:** Temporal Stability of facial skin microbiome for taxa with relative abundance ≥1% in at least one individual.

Phylum	Genus	Group-I (n = 24) Mean ± S.E.M	Group-II (n = 24) Mean ± S.E.M	p-value^1^ (Paired T test)
Actinobacteria	***All genera within phylum***	64.367 ± 4.795	67.199 ± 3.841	0.427
	Propionibacterium	56.232 ± 5.745	56.304 ± 5.748	0.980
	Corynebacterium	3.972 ± 0.875	6.690 ± 1.816	0.272
	Actinomyces	0.407 ± 0.100	0.205 ± 0.063	0.095
	Micrococcus	0.787 ± 0.276	0.382 ± 0.131	0.272
	f_Micrococcaceae_g	0.271 ± 0.088	0.108 ± 0.019	0.180
	Brevibacterium	0.356 ± 0.092	0.399 ± 0.077	0.750
	Kocuria	0.389 ± 0.091	0.164 ± 0.038	0.095
Proteobacteria	***All genera within phylum***	13.654 ± 2.175	8.860 ± 1.449	0.095
	**Paracoccus**	3.562 ± 0.657	1.116 ± 0.239	**3.3 × 10−4**
	f_Comamonadaceae_g	0.408 ± 0.219	0.118 ± 0.031	0.343
	Tepidimonas	0.002 ± 0.001	0.001 ± 0.001	0.467
	Neisseria	1.498 ± 0.425	1.025 ± 0.369	0.324
	Haemophilus	0.785 ± 0.260	0.435 ± 0.165	0.176
	Acinetobacter	1.431 ± 0.295	1.745 ± 0.460	0.589
	Lautropia	0.148 ± 0.102	0.034 ± 0.012	0.394
	f_Neisseriaceae_g	0.890 ± 0.374	0.798 ± 0.405	0.789
	f_Enterobacteriaceae_g	0.410 ± 0.224	0.054 ± 0.017	0.293
	f_Moraxellaceae_g	0.312 ± 0.125	0.302 ± 0.138	0.979
	Roseomonas	0.062 ± 0.043	0.006 ± 0.004	0.324
	f.Aeromonadaceae_g	0.205 ± 0.051	0.194 ± 0.074	0.937
	Actinobacillus	0.071 ± 0.060	0.015 ± 0.006	0.498
	Enhydrobacter	0.349 ± 0.056	0.499 ± 0.167	0.527
	Pseudomonadaceae_g	0.385 ± 0.087	0.191 ± 0.044	0.095
	Pseudomonas	0.361 ± 0.066	0.199 ± 0.038	0.095
Firmicutes	***All genera within phylum***	19.048 ± 2.539	21.260 ± 2.611	0.324
	Staphylococcus	9.681 ± 1.300	13.978 ± 2.205	0.108
	f_Ruminococcaceae_g	0.243 ± 0.159	0.027 ± 0.010	0.324
	Streptococcus	3.896 ± 0.944	2.381 ± 0.648	0.180
	Anaerococcus	0.907 ± 0.223	0.615 ± 0.159	0.210
	Peptoniphilus	0.326 ± 0.146	0.230 ± 0.076	0.502
	Bacillus	0.317 ± 0.068	0.177 ± 0.050	0.238
	f.Gemellaceae_g	0.228 ± 0.065	0.101 ± 0.027	0.095
	Granulicatella	0.314 ± 0.095	0.150 ± 0.038	0.143
	Lactobacillus	0.230 ± 0.060	0.297 ± 0.241	0.851
	o.Clostridiales;f__g	0.080 ± 0.045	0.015 ± 0.004	0.324
	SMB53	0.099 ± 0.071	0.018 ± 0.006	0.417
	f.Peptostreptococcaceae_g	0.079 ± 0.050	0.012 ± 0.003	0.324
	Veillonella	0.291 ± 0.070	0.130 ± 0.042	0.095
Bacteroidetes	***All genera within phylum***	1.451 ± 0.390	1.075 ± 0.472	0.427
	Porphyromonas	0.256 ± 0.127	0.085 ± 0.039	0.238
	Prevotella	0.345 ± 0.111	0.115 ± 0.051	0.095
	Capnocytophaga	0.180 ± 0.080	0.133 ± 0.074	0.675
Fusobacteria	***All genera within phylum***	0.38 ± 0.082	0.338 ± 0.128	0.817
Thermi	***All genera within phylum***	0.10 ± 0.017	0.090 ± 0.026	0.820

^1^p-value of paired T-test corrected for False Discovery Rate using Benjamini-Hochberg Method.
