# Supplementary material for: Sebum and Hydration Levels in Specific Regions of Human Face Significantly Predict the Nature and Diversity of Facial Skin Microbiome
Source: Sci Rep. 2016 Oct 27;6:36062. doi: 10.1038/srep36062 (PMC5081537; doi:10.1038/srep36062)
Supplement: Supplementary Information [file srep36062-s1.pdf]

Supplementary Material

Sebum and Hydration Levels in Specific Regions of Human Face Significantly Predict the Nature and Diversity of Facial Skin Microbiome

Souvik Mukherjee[1], Rupak Mitra[2], Arindam Maitra[3], Satyaranjan Gupta[2], Srikala Kumaran[2], Amit Chakrabortty[2] and Partha P. Majumder[3]

[1]BioMedical Genomics Centre, Kolkata, West Bengal, India, [2]Unilever R&D, Bangalore, Karnataka, India, [3]National Institute of Biomedical Genomics, Kalyani, West Bengal, India.

Supplementary Table 1. Initial QA/QC and removal of Chimeric Sequences from Raw Sequence Reads using QIIME

Sample ID	Average Length	Raw Sequence Reads	QA/QC filtered Reads	QA/QC failed Seqs	% QA/QC filtered	Non-Chimeric Reads	Chimeric Reads	% Chimeric Reads
3	518	62700	60951	1749	97.21	56406	4545	7.25
5	525	39003	36270	2733	92.99	35398	872	2.24
8	523	40478	38407	2071	94.88	36782	1625	4.01
9	511	31287	29873	1414	95.48	28887	986	3.15
11	522	112374	108641	3733	96.68	99974	8667	7.71
14	524	49955	47760	2195	95.61	45865	1895	3.79
15	528	29270	28166	1104	96.23	26780	1386	4.74
16	528	63046	61286	1760	97.21	59823	1463	2.32
25	530	38519	37041	1478	96.16	36339	702	1.82
26	515	41922	39840	2082	95.03	35304	4536	10.82
27	529	51542	49969	1573	96.95	48624	1345	2.61
28	519	78413	75278	3135	96.00	71457	3821	4.87
30	528	39484	37454	2030	94.86	36435	1019	2.58
32	528	92128	88175	3953	95.71	86288	1887	2.05
33	521	127476	122638	4838	96.20	99381	23257	18.24
40	520	46153	43867	2286	95.05	41323	2544	5.51
41	530	74532	70838	3694	95.04	69929	909	1.22
45	523	84225	80613	3612	95.71	76431	4182	4.97
46	524	111724	107938	3786	96.61	100706	7232	6.47
47	521	24157	22997	1160	95.20	20917	2080	8.61
49	527	20402	19450	952	95.33	19064	386	1.89
50	520	47712	45337	2375	95.02	43205	2132	4.47
51	528	27148	26206	942	96.53	26040	166	0.61
57	524	99521	95494	4027	95.95	85332	10162	10.21
61	513	80577	75993	4584	94.31	70006	5987	7.43
62	523	47557	45559	1998	95.80	43027	2532	5.32
67	522	28220	27218	1002	96.45	25841	1377	4.88
78	530	72817	70671	2146	97.05	69502	1169	1.61
83	528	38266	36108	2158	94.36	36024	84	0.22

Supplementary Table 2. Comparison of Taxonomic Classification Data at the Phyla and Genera Levels identified by Mothur and QIIME analyses

Classifier	Database	Phyla Level Classification					Genera Level Classification				
		Classified Bacterial Phyla	Unclassified (Cumulative Average of % Proportions)	Archaebacteria	Common Phyla (Cumulative Average of % Proportions)	Uniquely Identified Phyla (Cumulative Average of % Proportions)	Classified Bacterial Genera	Unclassified (Cumulative Average of % Proportions)	Archaebacteria	Common Genera (Cumulative Average of % Proportions)	Uniquely Identified Genera (Cumulative Average of % Proportions)
QIIME (v.1.8.0)	Greengenes	41	3 (1.04%)	1	26 (98.95%)	15 (0.01%)	610	536 (10.5%)*	2	456 (88.8%)	153 (0.7%)
Mothur (v.1.33.3)	Silva Gold	26	2 (0.6%)	1	26(99.4%)	0	863	177 (7.3%)	2	456 (88.7%)	407 (4%)*

* The percentage relative abundance is estimated including Streptophyta with average % value of 2.5%

Supplementary Table 3. Comparative Study with Skin Microbiome Profiles in Other Populations

**Tax. Level**	**Name of Bacterial Taxa**	**Zeeuwen*et al* 2012 (%)**	**Our Study (%)**
**Phylum**	**Proteobacteria**	**0.43**	**13.06**
	Firmicutes	11.67	17.72
	Actinobacteria	86.82	66.26
**Genus**	Finegoldia	0.43	0.16
	**Streptococcus**	**0.75**	**3.99**
	Staphylococcus	9.42	8.63
	Propionibacterium	76.01	58.60
	**Corynebacterium**	**7.65**	**3.61**

Supplementary Figure 1. Correlation Plots between Sebum and Hydration levels of (a) Forehead and (b) Cheek regions of the human face

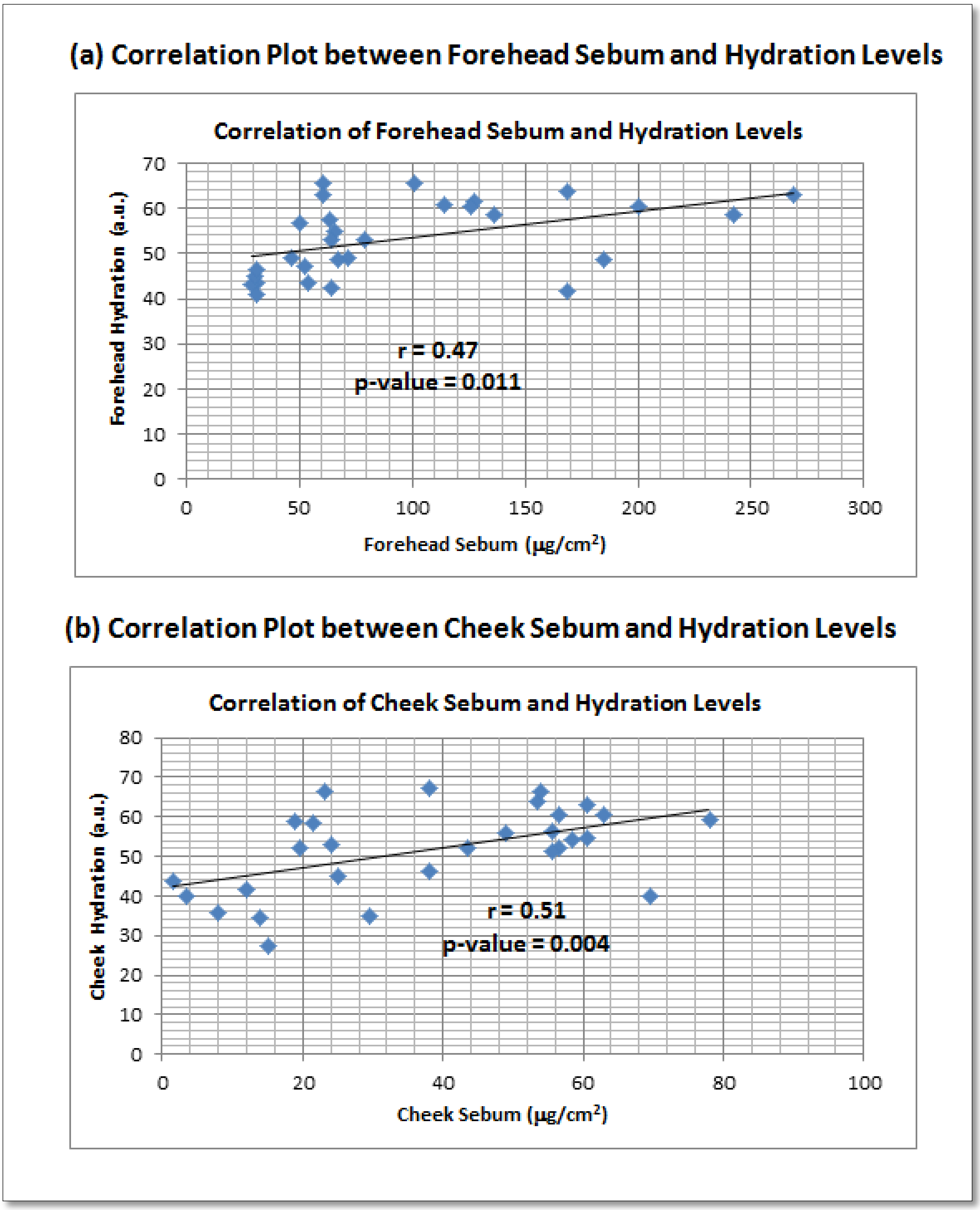

Supplementary Figure 2. Rarefaction plots with subsampling for minimum number of sequence reads

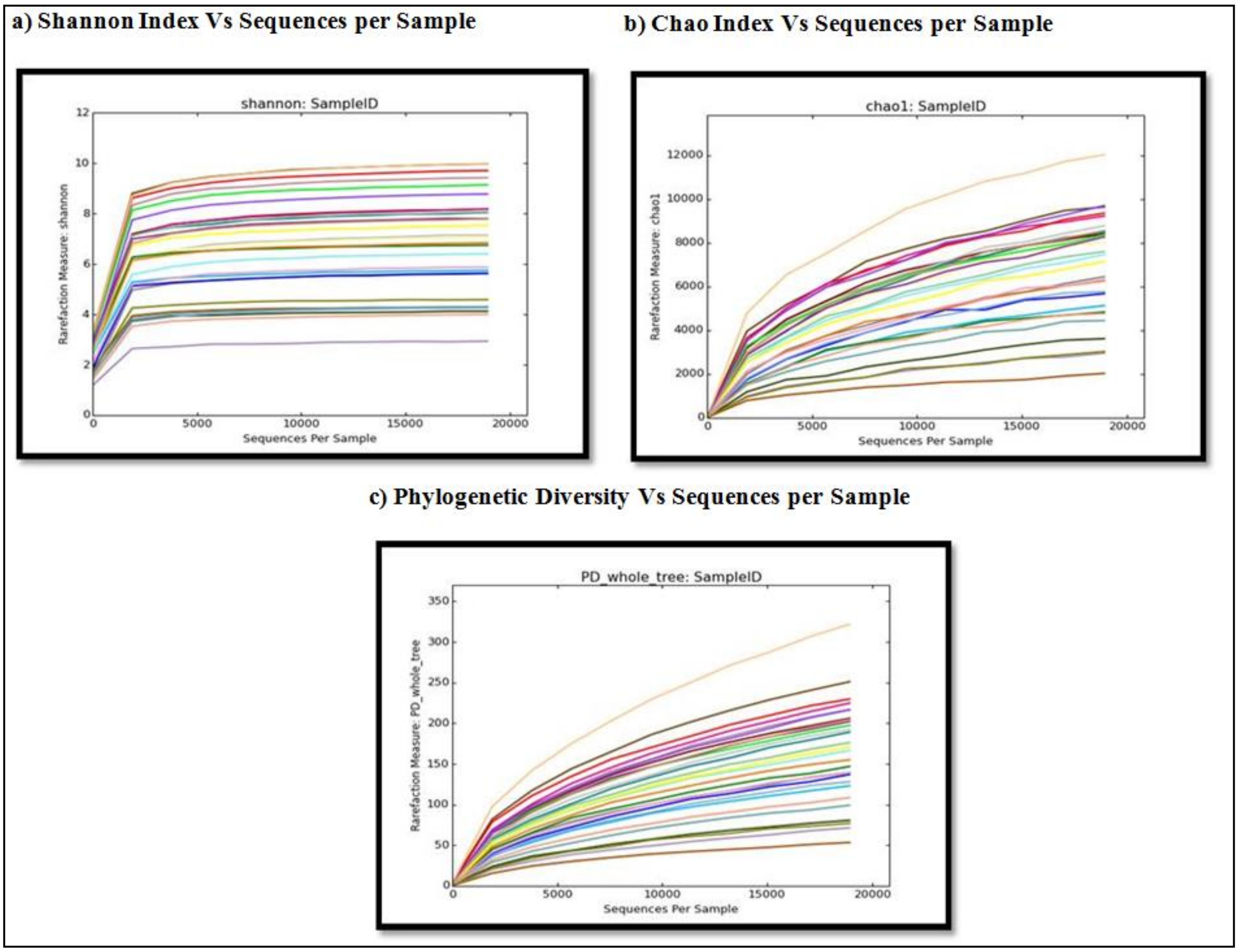

Supplementary Figure 3. Correlation Plot between Shannon and Chao Indices in all the 29 individuals

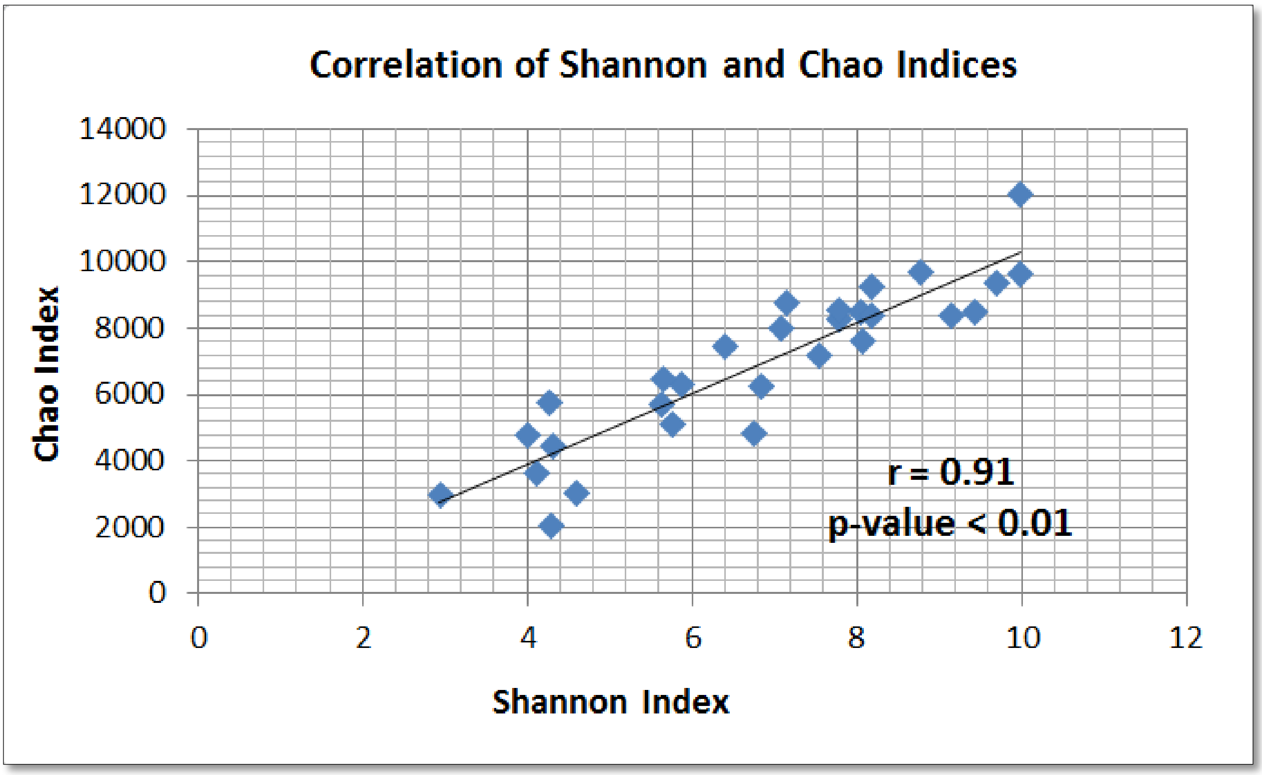

Supplementary Figure 4. Inter-Individual variability in number of Operational Taxonomic Units (OTUs) with subsampling for minimum number of sequence reads

(The numbers on top of individual histograms are the total number of OTUs for that individual)

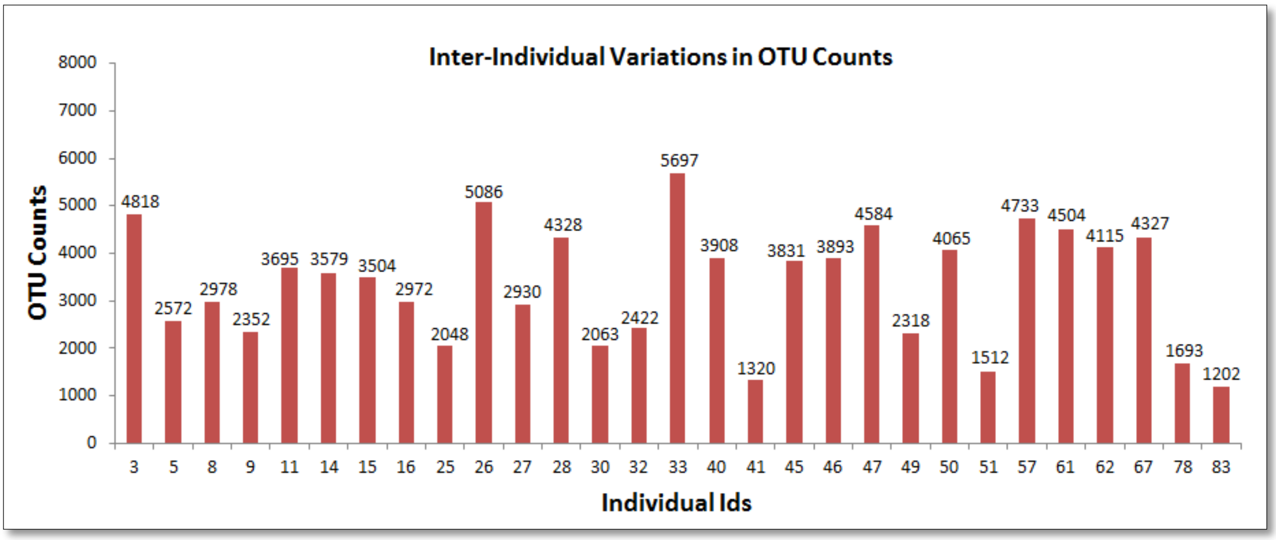

Supplementary Figure 5. Correlation Plot of Number of Non-Chimeric Sequence Reads with Number of OTUs

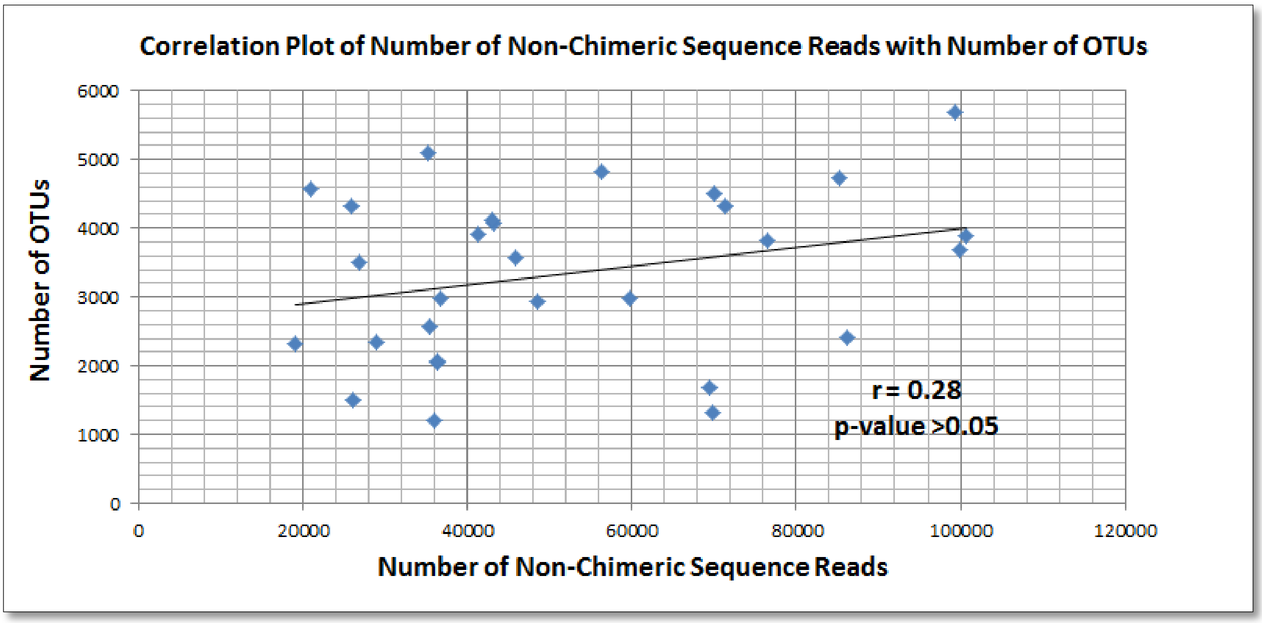

Supplementary Figure 6. Correlation Plots between bacterial abundance derived from Mothur and QIIME for Common Phyla with Average Relative abundance (a) >1% and (b) ≤ 1% identified by both classifiers

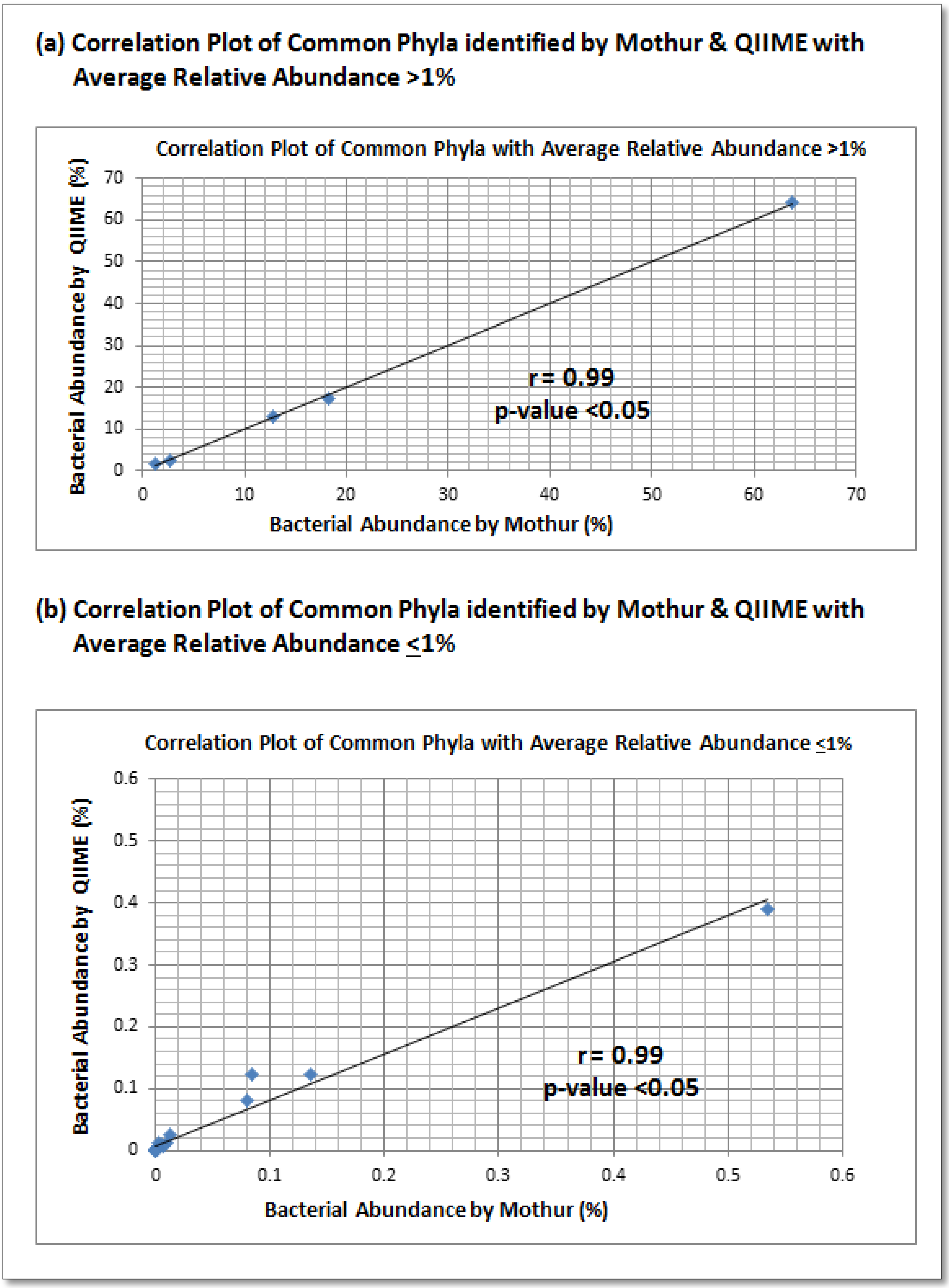

Supplementary Figure 7. Correlation Plots between bacterial abundance derived from Mothur and QIIME for Common Genera with Average Relative abundance (a) >1% and (b) ≤ 1% identified by both classifiers

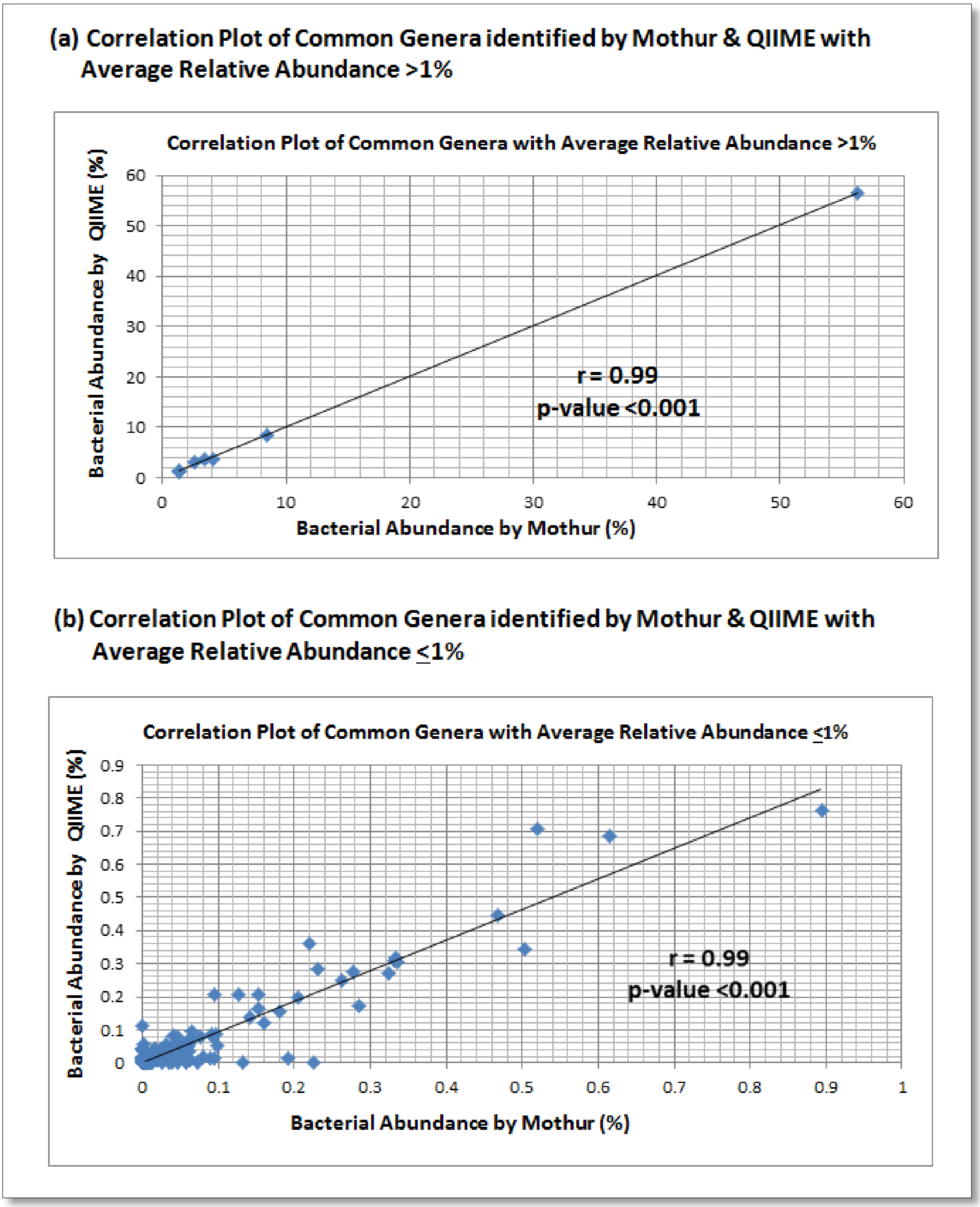

Supplementary Figure 8. Weighted Unifrac Plots displaying beta-diversity measures of inter-individual microbiome profiles

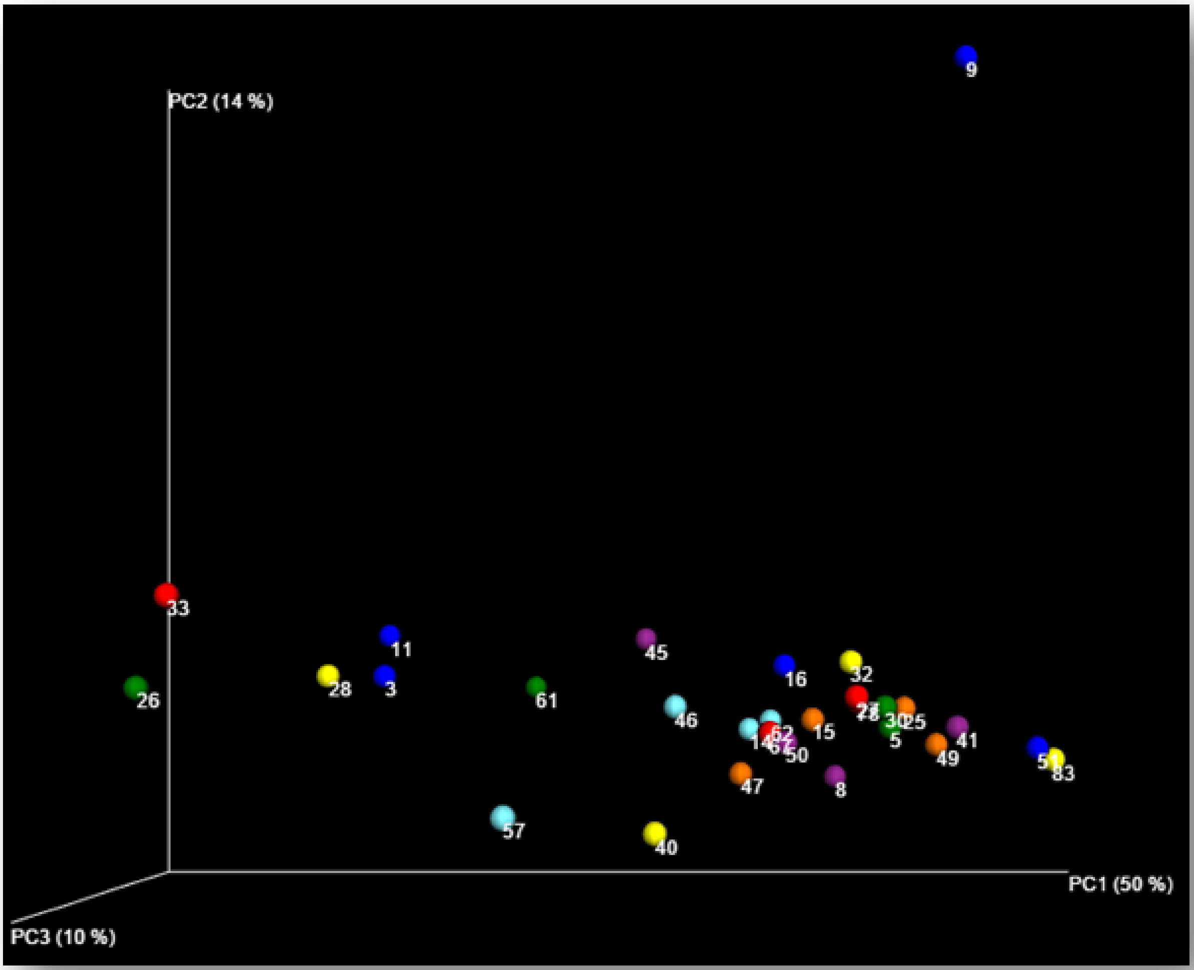

Colours in the figure denote the barcodes used for multiplexed sequencing

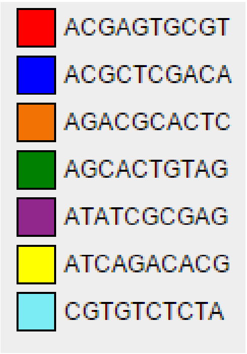

Supplementary Figure 9. OTU Heatmap of Major OTUs with ≥ 1000 sequence reads along with their Taxonomic Classifications

(The numbers within the heatmap are the actual number of reads for individual samples. The taxonomic classifications are given in the right side)

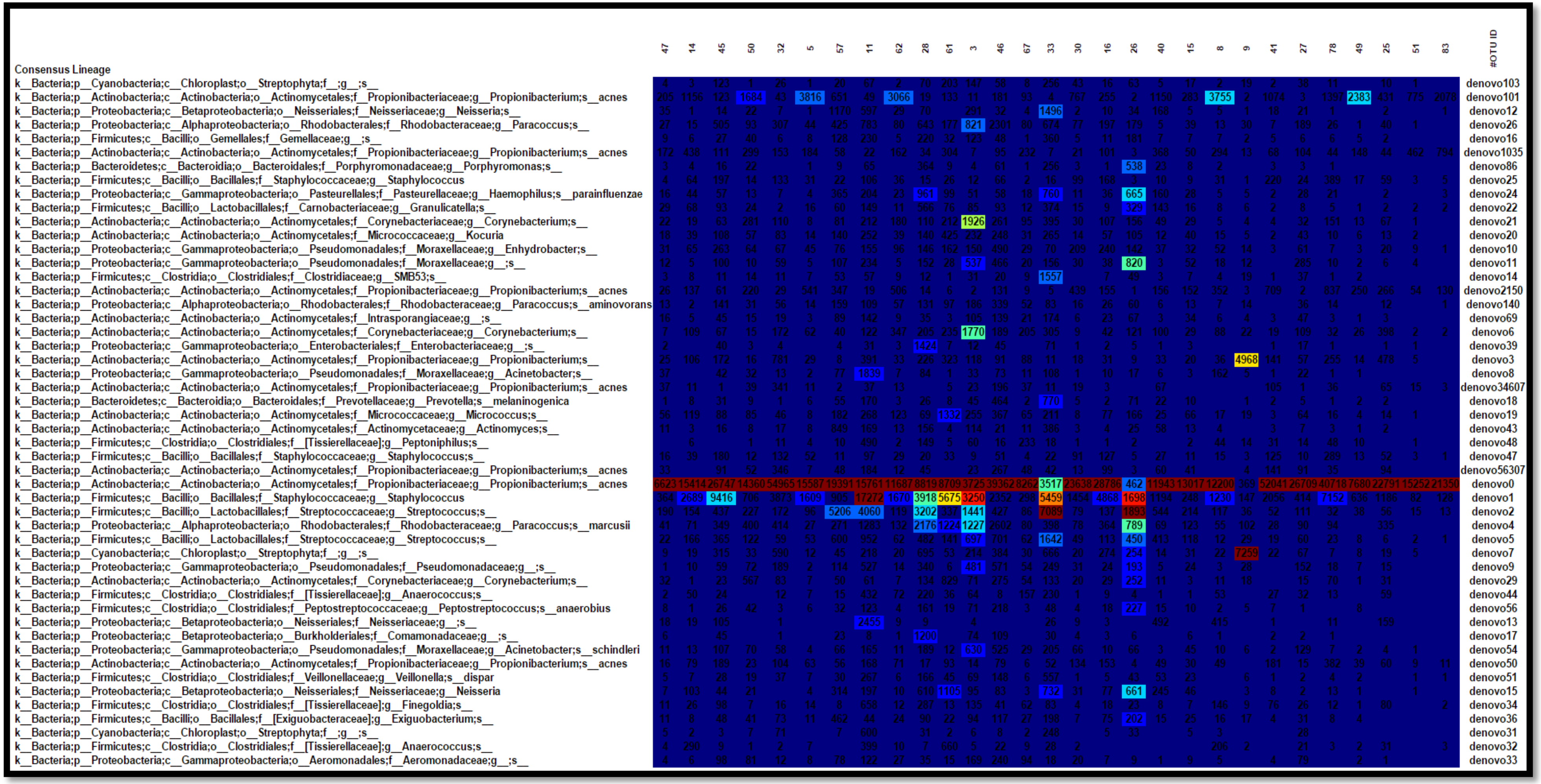

Supplementary Figure 10. Gel Electrophoresis image for V3 PCR amplification using bacterial DNA isolated from individual facial swabs and the negative control cotton swab

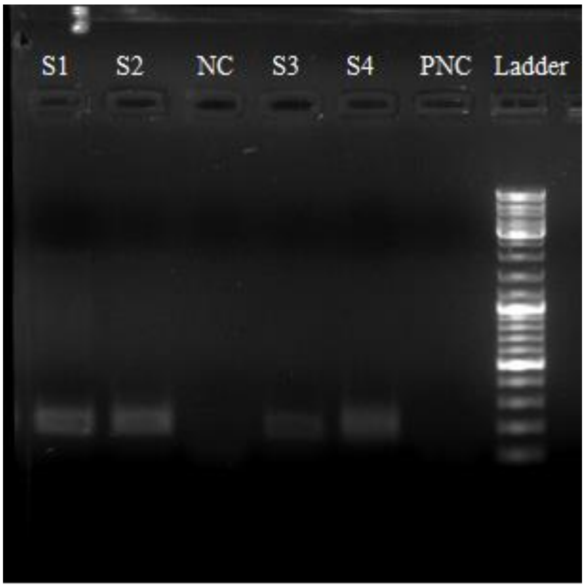

S1, S2, S3 & S4 are individual samples with individual ids: 3, 16, 27 and 28 respectively

NC = Negative Control Cotton Swab soaked in PBS buffer

PNC = PCR Mix Negative Control

Ladder = 100-10000 bp DNA Ladder (Gene Ruler DNA Ladder Mix)

V3 specific Primers used:
16S V3 FW: CCTACGGGAGGCAGCAG
16S V3 RV: ATTACCGCGGCTGCTGG
Fragment Size = ~200 bp

Sl.No.	Sample No.	Qubit Concn. of DNA isolated from Cotton Swab (ng/µl)	Qubit Concn. after PCR & before gel purification (ng/µl)	Qubit Concn. after gel purification (ng/µl)
S1	3	0.22	N.A.[1]	9.49
S2	16	0.18	N.A.[1]	7.92
S3	27	0.14	N.A.[1]	9.59
S4	28	0.10	N.A.[1]	8.65
NC	Negative Control Cotton Swab	Below Detectable Range	0.74	Below Detectable Range
PNC	PCR Negative Control	Below Detectable Range	Below Detectable Range	Below Detectable Range

[1]Qubit concentrations of samples were estimated only after gel purification
